# Lithium treatment reverses irradiation-induced changes in rodent neural progenitors and rescues cognition

**DOI:** 10.1038/s41380-019-0584-0

**Published:** 2019-11-14

**Authors:** Giulia Zanni, Shinobu Goto, Adamantia F. Fragopoulou, Giulia Gaudenzi, Vinogran Naidoo, Elena Di Martino, Gabriel Levy, Cecilia A. Dominguez, Olga Dethlefsen, Angel Cedazo-Minguez, Paula Merino-Serrais, Antonios Stamatakis, Ola Hermanson, Klas Blomgren

**Affiliations:** 1 Department of Women’s and Children’s Health, Karolinska Institutet, BioClinicum J9:30, 171 64 Stockholm, Sweden; 2 Department of Developmental Neuroscience, New York State Psychiatric Institute, Columbia University, 1051 Riverside, New York, NY 10032 USA; 3 Department of Obstetrics and Gynecology, Nagoya City University Graduate School of Medical Sciences, 467-8601, 1, Kawasumi, Mizuho-cho, Mizuho-ku, Nagoya, Japan; 4 Department of Neuroscience, Karolinska Institutet, Biomedicum, 171 77 Stockholm, Sweden; 5 Department of Protein Science, Division of Nanobiotechnology, KTH Royal Institute of Technology, Science for Life Laboratory, 171 21 Stockholm, Sweden; 6 Department of Human Biology, Faculty of Health Sciences, Anzio Road Observatory, 7925, University of Cape Town, Cape Town, South Africa; 7 Ludwig Institute for Cancer Research, Brussels Branch, Avenue Hippocrate 75, 1200 Brussels, Belgium; 8 National Bioinformatics Infrastructure Sweden (NIBIS), Science for Life Laboratory (SciLifeLab), Svante Arrhenius väg 16C, 106 91 Stockholm, Sweden; 9 Department of Biochemistry and Biophysics (DBB), Stockholm University, Svante Arrhenius väg 16C, 106 91 Stockholm, Sweden; 10 Department of Neurobiology, Care Sciences and Society, Center for Alzheimer Research, Division of Neurogeriatrics, Karolinska Institutet, BioClinicum J9:20, 171 64 Stockholm, Sweden; 11 Biology-Biochemistry Lab, Faculty of Nursing, School of Health Sciences, National and Kapodistrian University of Athens, Papadiamantopoulou 123, Goudi, 11527 Athens, Greece; 12 Pediatric Oncology, Karolinska University Hospital, Eugeniavägen 23, 171 64 Stockholm, Sweden

**Keywords:** Neuroscience, Diseases

## Abstract

Cranial radiotherapy in children has detrimental effects on cognition, mood, and social competence in young cancer survivors. Treatments harnessing hippocampal neurogenesis are currently of great relevance in this context. Lithium, a well-known mood stabilizer, has both neuroprotective, pro-neurogenic as well as antitumor effects, and in the current study we introduced lithium treatment 4 weeks after irradiation. Female mice received a single 4 Gy whole-brain radiation dose on postnatal day (PND) 21 and were randomized to 0.24% Li2CO_3_ chow or normal chow from PND 49 to 77. Hippocampal neurogenesis was assessed on PND 77, 91, and 105. We found that lithium treatment had a pro-proliferative effect on neural progenitors, but neuronal integration occurred only after it was discontinued. Also, the treatment ameliorated deficits in spatial learning and memory retention observed in irradiated mice. Gene expression profiling and DNA methylation analysis identified two novel factors related to the observed effects, Tppp, associated with microtubule stabilization, and GAD2/65, associated with neuronal signaling. Our results show that lithium treatment reverses irradiation-induced loss of hippocampal neurogenesis and cognitive impairment even when introduced long after the injury. We propose that lithium treatment should be intermittent in order to first make neural progenitors proliferate and then, upon discontinuation, allow them to differentiate. Our findings suggest that pharmacological treatment of cognitive so-called late effects in childhood cancer survivors is possible.

## Introduction

Dramatic improvements in childhood cancer survival rates have been made in the last decades [1] due to the great strides in the treatments. The treatments encompass a combination of surgery, chemotherapy, and radiotherapy with the recent addition of immunotherapy. However, the growing population of survivors often has to face therapy-related morbidity [2]. Radiotherapy is known to cause debilitating cognitive alterations [3] leading to impaired processing speed, attention and working memory, leading to social isolation, further impinging on emotional and psychological well-being and ultimately leading to anxiety, and posttraumatic stress symptoms [4]. Declines in IQ and academic achievements have been observed during longitudinal follow-up and these impact the quality of life, academic performance, and overall daily activities [5, 6].

Different mechanisms have been implicated in the cognitive changes observed in patients treated with radiotherapy [7]. Increased levels of cytokines seem to mediate some of these changes, as well as direct and indirect DNA damage, endocrine dysfunction, activation of microglia and astrocytes, hypomyelination, and decreased neurogenesis [5, 8, 9]. In particular neurogenic regions, harboring cellular proliferation, display higher sensitivity to irradiation as seen in rodent models and in humans [10–12]. Irradiation, even after a single moderate dose, was shown to cause apoptosis and progressive decline in neurogenesis of young rats and mice resulting in severe cognitive declines [10, 13–17]. Adult hippocampal neurogenesis persists throughout life, mainly in the subgranular zone (SGZ) of the hippocampus and the subventricular zone of the lateral ventricles (Altman and Das 1965). These regions harbor neural stem and progenitor cells (NSPCs) dividing continuously, giving birth to newborn neurons. This is believed to contribute to hippocampal plasticity and especially learning, memory and mood regulation [18, 19].

Lithium, commonly used in the treatment of bipolar disorder, has been shown to exert neuroprotective and regenerative effects in a variety of neurological insults [20]. In preclinical studies lithium protected the neonatal brain against the neurodegenerative effects of hypoxia-ischemia (HI) [21–23] and rescued cognitive loss in adult as well as in young mice after cranial irradiation [24–26]. The neuroprotective effects of lithium after cranial irradiation are attributable to enhanced hippocampal neurogenesis and decreased apoptosis in young rats and mice [24, 25].

Lithium also restored synaptic plasticity in a Down syndrome mouse model [27] and ongoing trials aim at introducing lithium as a treatment of a broad range of brain-related disorders [28]. Despite the surge of studies conducted on lithium, the exact mechanisms of action are only partly elucidated. Lithium exerts its action through the modulation of intracellular second messengers with subsequent alteration of complex and interconnected intracellular enzyme cascades [29]. One target is the protein kinase glycogen synthase kinase Gsk3β [30]. Direct [31, 32] and indirect inhibition of Gsk3β by lithium [33] leads to improvements of impaired cognition, likely involving a variety of different mechanisms, such as supporting long-term potentiation and diminishing long-term depression, promotion of neurogenesis and ultimately reduction of inflammation and apoptosis [24, 25, 34, 35].

Encouraging results support the use of lithium in combination with cancer treatment to improve the therapeutic effect, for example as a radiosensitizer [36–39]. Nevertheless, a post radiotherapy lithium treatment may still be preferable to safely exclude the risk of protecting tumor cells, thus increasing the risk of relapses. The study herein investigates the effects of lithium treatment on NSPC proliferation, survival, dendritic orientation, and cognition after brain irradiation, and whether therapeutically relevant doses can rescue neurogenesis and ameliorate memory deficits even long after irradiation of the juvenile brain. Ultimately, we provide evidence for a possible molecular mechanism involving novel proteins targeted by lithium and further elucidate the effects of irradiation on fate commitment of NSPCs and how lithium can harness this process.

## Results

**Fig. 1:**
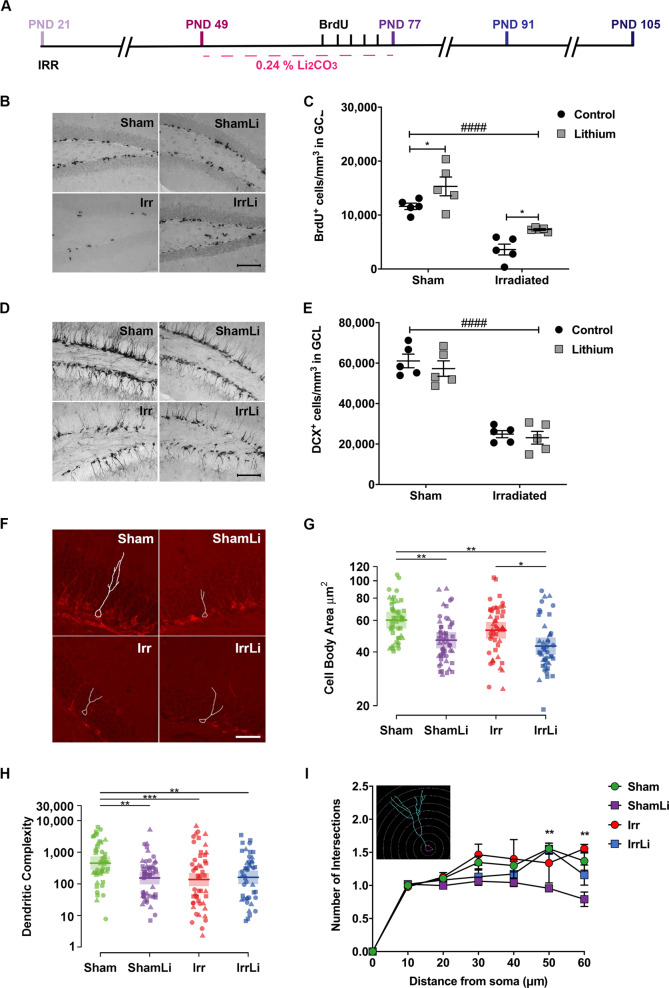
Lithium treatment 4 weeks after irradiation increased proliferation but not neuronal maturation in the GCL on PND 77. **a** Timeline of the in vivo experimental design showing that lithium treatment was started 4 weeks after irradiation; three different time points (PND 77, 91, and 105) were investigated to assess the early as well as late effects of lithium on neurogenesis. **b** BrdU immunoreactivity in the GCL of the different treatment groups at PND 77. Scale bar = 100 µm. ×20 magnification. **c** Interleaved dot plot graph of BrdU quantification in GCL showing the effect of lithium treatment in the Sham (*p = 0.0485) and Irr groups (*p = 0.0471). Two-way ANOVA shows that both irradiation and lithium treatments had an effect: irradiation (*F*_1,16_ = 57.77, ^####^*p* < 0.0001), lithium (*F*_1,16_ = 12.45, **p = 0.0028). **d** DCX immunoreactivity in the GCL of the different treatment groups on PND 77. Scale bar = 100 µm. ×20 magnification. **e** Interleaved dot plot graph of DCX quantification in the GCL on PND 77. Two-way ANOVA revealed an effect of irradiation but not lithium treatment: irradiation (*F*_1,16_ = 127.3, ^*####*^*p* < 0.0001), lithium (*F*_1,16_ = 1.34, *p* = 0.7974). Lithium treatment did not alter the density of DCX-positive cells in Sham (*p* = 0.8097) or Irr (*p* > 0.9999) groups. **f** Representative images of the dendritic tracing in DCX^+^ cells for all experimental groups. **g** Cell body area (µm^2^) shows that lithium treatment significantly decreased the size of the immature cells both in Sham and Irr groups at PND 77. **h** Dot plot graph of the dendritic complexity of DCX^+^ immature neurons showing lower complexity in ShamLi, Irr, and IrrLi compared with Sham at PND 77. Circle, triangle, and square marks represent the value for each neuron in each animal. *N* (number of animals) = 3 in each group. Number of traced neurons is 15–20 in each animal. Linear mixed model, *p *<* 0.05, **p *<* 0.01, ***p *<* 0.001. Error bars represent point estimate and 95% confidence interval. **i** Sholl analysis of DCX^+^ immature neurons showing that lithium treatment reduced the number of intersections in the distal part (50–60 µm) of the dendrites at PND 77 in Sham but not in Irr brains. Error bars represent SEM ****p < 0.0001; **p < 0.01; *p < 0.05

Assessment of neurogenesis (proliferation, dendritic orientation, and survival of the NSPCs) was performed at three different time points, immediately after (PND 77), 2 weeks after (PND 91) and 4 weeks after (PND 105) the termination of lithium chow exposure (Fig. 1a in vivo study design).

### Four weeks of continuous lithium treatment increased the number of proliferating cells in the DG but decreased the cell body area and the dendritic complexity of doublecortin-positive (DCX^+^) cells

To analyze the effect of lithium on proliferation of NSPCs, we measured BrdU incorporation on PND 77 (Fig. 1b). Proliferation in the GCL was decreased significantly by irradiation in both vehicle and lithium-treated mice (Fig. 1c). Lithium increased the density of proliferating cells in both sham and irradiated brains, 83% in the irradiated group and 33% in the sham group. We further determined the effects of lithium on the density of doublecortin-positive (DCX^+^) cells in the GCL on PND 77 (Fig. 1d). Irradiation provoked a 60% decrease of the DCX^+^ cell density in the GCL, but lithium had no effect on DCX^+^ cell density, neither in sham, nor in irradiated brains (Fig. 1e).

In addition, we conducted morphometric analysis of DCX^+^ immature neurons on PND 77 (Fig. 1f). We found that lithium treatment decreased the cell body area in both sham and irradiated animals (Fig. 1g), whereas radiation drastically decreased the dendritic complexity. Reduced dendritic complexity was observed in ShamLi, Irr, and IrrLi groups on PND 77 (Fig. 1h). Sholl analysis showed that lithium treatment reduced the number of intersections at the distal part of the dendrites (50 and 60 μm from the soma) on PND 77 in the sham, but not in irradiated animals (Fig. 1i).

### Lithium discontinuation prevented irradiation-induced alterations in process orientation and dendritic complexity of DCX^+^ cells on PND 91

**Fig. 2:**
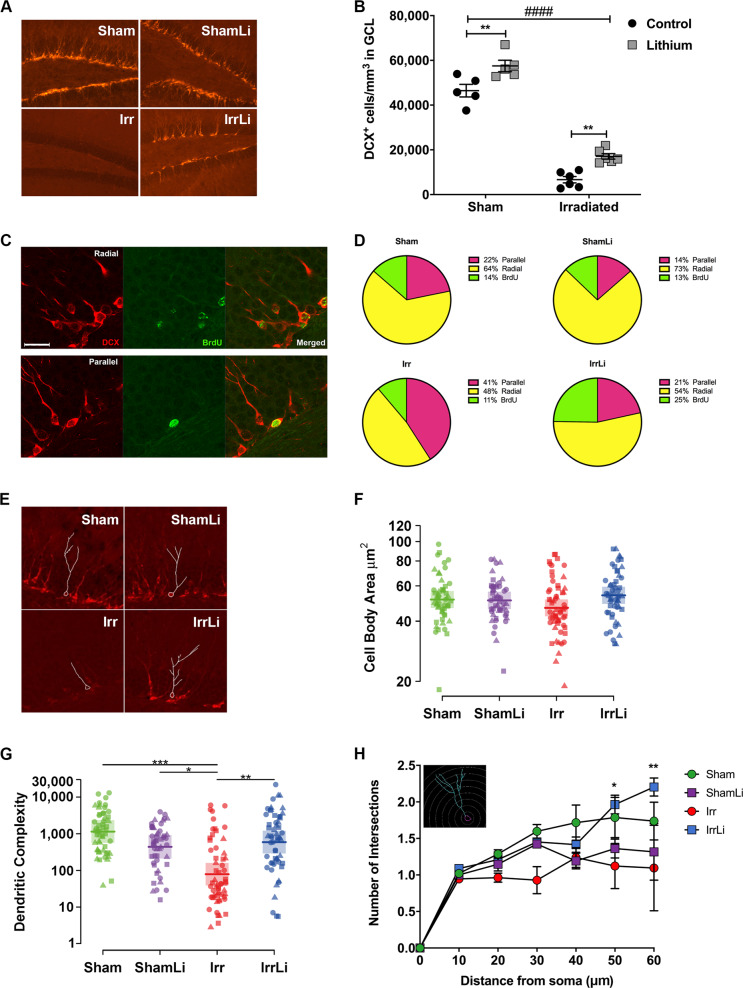
Lithium restored irradiation-induced alterations in dendrite arborization of doublecortin (DCX^+^) cells in the subgranular zone (SGZ) of the DG at PND 91. **a** DCX immunoreactivity in the GCL of the different treatment groups at PND 91. Scale bar = 100 µm. ×20 magnification. **b** Interleaved bar graph of the quantification of DCX^+^ cells in the GCL showing the effect of lithium treatment in the Sham group (**p = 0.0030) and in the Irr group (**p = 0.0023). Two-way ANOVA shows the effect of both treatments: irradiation (*F*_1,18_ = 400.6, ^####^*p* < 0.0001), lithium (*F*_1,18_ = 28.70, ****p < 0.0001). **c** The phenotype of the radial (upper panel) and parallel (lower panel) processes in immature neurons at PND 91 was determined by double labeling for BrdU and DCX as shown in the representative confocal images of DCX+ (red), BrdU^+^ (green) and merged BrdU^+^–DCX^+^ cells in the GCL. Scale bar = 20 µm. **d** Pie chart of the percentages of BrdU cells double labeled for DCX and with either radial or parallel process phenotype. The percentages of radial processes were not affected by lithium treatment: in the Sham group *p* = 0.2341 and in the Irr group *p* = 0.5313. Two-way ANOVA shows the effect of irradiation on radial processes: irradiation (*F*_1,18_ = 24.4, ***p *=* 0.0001), lithium (*F*_1,18_ = 3.96, *p* *=* 0.0619). The percentages of parallel processes were increased after irradiation, and lithium treatment restored the percentages of parallel processes in the Irr group (****p < 0.0001) but did not affect the Sham group (*p* = 0.0570). Two-way ANOVA: irradiation (*F*_1,18_ = 33.59, ****p < 0.0001), lithium (*F*_1,18_ = 34.75, ****p < 0.0001). The percentages of BrdU not labeled for DCX were not affected by irradiation but were increased in the IrrLi group (**p = 0.0094). Two-way ANOVA: interaction (*F*_1,18_ = 5.277, *p = 0.0338). **e** Representative images of the dendritic tracing in DCX^+^ cells in animals for all experimental groups. **f** Dot plot graph of the cell body area showing no effect of either lithium or irradiation at PND 91. **g** Dot plot graph of the dendritic complexity of DCX^+^ immature neurons showing significant decrease in the Irr group compared with all the other groups at PND 91. Circle, triangle and square marks represent the value for each neuron in each animal. *N* (number of animals) = 3 in each group. Number of traced neurons is 15–20 in each animal. Linear mixed model, *p < 0.05, **p < 0.01, ***p < 0.001. Error bars represent point estimate and 95% confidence interval. **h** Scholl analysis of DCX^+^ immature neurons showing that lithium treatment restores the number of intersections in the distal part (50–60 µm) of the dendrites at PND 91 in the Irr but not in the Sham-treated group. *N* (number of animals) = 3 in each group. Number of traced neurons is 15–20 in each animal. Two-way ANOVA and post hoc Bonferroni test. *p < 0.05, ***p < 0.001 compared with the Irr group. Error bars represent SEM. ****p < 0.0001; **p < 0.01; *p < 0.05

To assess if the increase in proliferating cells on PND 77 resulted in increased survival and differentiation into immature neurons, we analyzed the density of DCX^+^ cells in the GCL on PND 91, 2 weeks after lithium discontinuation (Fig. 2a). DCX^+^ cell density was decreased by irradiation, as expected, but this decrease was reversed by lithium treatment. Also, in nonirradiated brains the DCX^+^ cell density increased. The increase was 156% and 24% in the irradiated and sham lithium-treated groups, respectively (Fig. 2b). To further address the effects of irradiation and lithium treatment on the integration of newly born neurons, we performed a DCX^+^/BrdU^+^ double immunostaining (Fig. 2c), enabling us to determine if the orientation of the main dendritic process of neurons born during the last 5 days of lithium treatment was parallel or radial to the GCL. We have earlier shown that irradiation causes the main dendritic process to shift from a radial to a parallel orientation [40]. Our results confirmed that irradiation increased the percentage of parallel processes from 22% in sham to 41% in irradiated, whereas the number of radial processes was decreased from 64% in sham to 48% in irradiated (Fig. 2d). Remarkably, we found that lithium treatment reduced the percentage of parallel main processes by half, from 41 to 21%, in the irradiated brains, to the same level as in the sham controls (22%) (Fig. 2d). However, lithium did not alter the proportion of radial main processes, neither in the sham nor in the irradiated brains. Also, we found that lithium treatment increased the number of BrdU^+^ cells that were not labeled for DCX in the irradiated group (25%) compared with the irradiated group not treated with lithium (11%). However, the morphometric analysis of the DCX^+^ cells on PND 91 (Fig. 2e) revealed that the cell body area of the irradiated groups was not different from the sham groups, and lithium did not affect cell body area either (Fig. 2f). The dendritic complexity of the DCX^+^ cells in the irradiated group was drastically decreased compared with the sham group (7%), but lithium treatment restored this completely to the sham level (Fig. 2g). This was further confirmed by Sholl analysis, where we found that the number of intersections at 50 and 60 µm from the soma was normalized to sham level in the irradiated group treated with lithium on PND 91 (Fig. 2h).

### Lithium-upregulated genes involved in cell cycle and neuronal signaling after irradiation in vitro—association with changes in DNA methylation

**Fig. 3:**
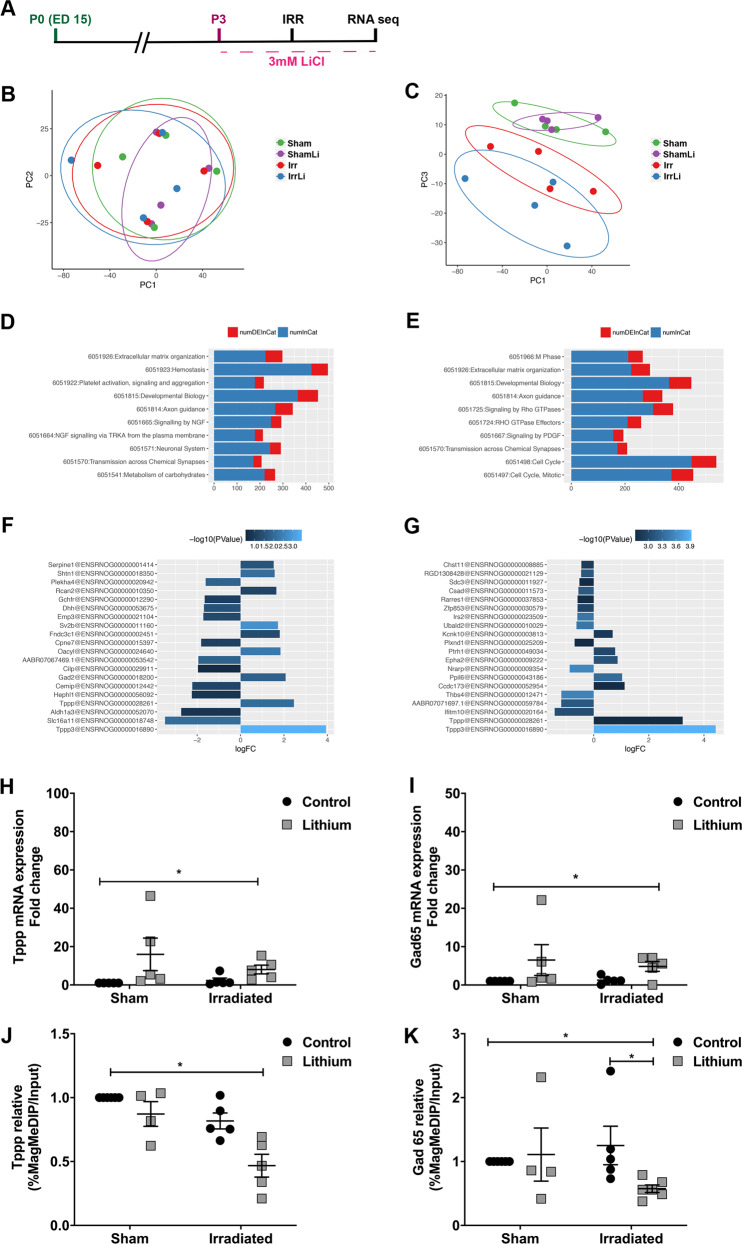
Lithium regulated the expression of genes involved in cell cycle and neuronal transmission in vitro. **a** Timeline of the in vitro experimental design showing that neural stem progenitor cells (NSPC) were established at passage 0 (P0) and at passage 3 (P3) they were exposed to lithium and irradiation followed by RNA sequencing. **b, c** Principal component (PC) analysis plots based on the filtered and normalized counts per million that allowed us to identify the uncorrelated variables to explain the source of maximum amount of variance in the different treatment groups (Sham, ShamLi, Irr, and IrrLi). **d** Gene ontology (GO) analysis revealed that the changes in the reactome of different treatment groups for PC2 were related to neuronal transmission. ‘numDEInCat’ is the number of differentially expressed (DE) genes in the corresponding GO category, whereas ‘numInCat’ is the number of detected genes in the corresponding GO category. **e** Gene ontology (GO) analysis revealed that the changes in the reactome of different treatment groups for PC3 were related to cell cycle regulation. **f** Table of differentially expressed genes in Sham cells compared with ShamLi cells treated with lithium. **g** Table of differentially expressed genes in irradiated cells compared with irradiated cells. **h** Gene expression analysis of the identified cell cycle gene shows that lithium significantly increased Tppp expression in both sham and irradiated cells. Two-way ANOVA shows the effect of lithium treatment: irradiation (*F*_1,16_ = 0.5473, *p* = 0.4702), LiCl (*F*_1,16_ = 5.439, *p = 0.0331). **i** Gene expression analysis of the identified neuronal transmission gene shows that lithium significantly increased GAD65 expression in both Sham and Irr cells. Two-way ANOVA shows the effect of lithium treatment: irradiation (*F*_1,16_ = 0.1073, *p* = 0.7475), LiCl (*F*_1,16_ = 4.576, *p = 0.0482). Error bars represent SEM. ****p < 0.0001; **p < 0.01; *p < 0.05. **j** Methylation analysis by MeDIP-qPCR of *Tppp* showing a significant difference between groups (*p = 0.0145). Post hoc test showing a significant decrease in the IrrLi group compared with the Sham group (*p *=* 0.0123). **k** MeDIP-qPCR analysis of Gad65 showing a significant difference between groups (*p *=* 0.0112). Post hoc test showing a significant decrease in the IrrLi group compared with the Sham (*p *=* 0.0284) and IrrLi groups (*p *=* 0.0273)

In order to investigate the molecular mechanisms possibly involved in the lithium effects after irradiation, we designed an in vitro study (Fig. 3a) where isolated NSPCs from the telencephalon of 15.5 days’ rat embryos, after expansion passage 3 (P3) were exposed to 2.5 Gy irradiation and lithium treatment [41]. Initially, we performed RNA sequencing and compared the Reactome across different conditions. Principal component analysis revealed that the sham groups clustered together for PC2 (Fig. 3b) whereas the irradiated groups clustered for PC3 (Fig. 3c), indicating that NSPCs responded in a specific manner to the individual treatments. A deeper analysis of the Reactome profile with respect to each PC revealed that PC2 identified changes in gene expression related to a variety of brain developmental as well as synaptic transmission processes (Fig. 3d), whereas PC3 identified differential gene expression for genes involved primarily in cell cycle as well as axonal guidance (Fig. 3e). To identify individual candidate genes involved in the specific responses, we next looked for the highest fold changes in gene expression between sham and sham treated with lithium (Fig. 3f) and between irradiated and irradiated treated with lithium (Fig. 3g). This analysis highlighted two genes, one that encodes for tubulin polymerization-promoting protein (Tppp) and glutamate decarboxylase 2 (*GAD2*) gene that encodes for the GAD65 protein [42]. The mRNA levels were confirmed by RT-qPCR and lithium accounted for most of the variability in the sham and irradiated groups in *Tppp* expression levels (Fig. 3h) as well as in GAD65 (Fig. 3i).

To investigate possible mechanisms underlying the changes in gene expression, we next investigated the effects on DNA methylation of the regulatory regions of *Tppp* and the *GAD2* gene, using a MeDIP-based approach [43] and analyzing the 5-methylcytosine (5mc) levels of the regulatory regions of these two genes in the in vitro fetal neural stem cell model we have developed. DNA methylation of regulatory regions is most often, but not exclusively, associated with transcriptional repression, and decreased levels of methylation are thus most often, but not always, associated with increased and high levels of gene expression [44]. These experiments revealed a clear correlation between the effects of irradiation and lithium treatment on gene expression and the corresponding DNA methylation levels. At the *Tppp* gene, 5mc levels were significantly decreased after lithium treatment of irradiated cells compared with the sham control (Fig. 3j). The 5mc levels at the *GAD2* regulatory region also showed a significant decrease in the irradiated group treated with lithium compared with sham control and irradiated groups (Fig. 3k). These results suggest that lithium treatment after irradiation influences epigenetic mechanisms yielding decreased DNA methylation levels.

### Lithium upregulated Tppp and GAD65 levels in the irradiated mice in vivo

**Fig. 4:**
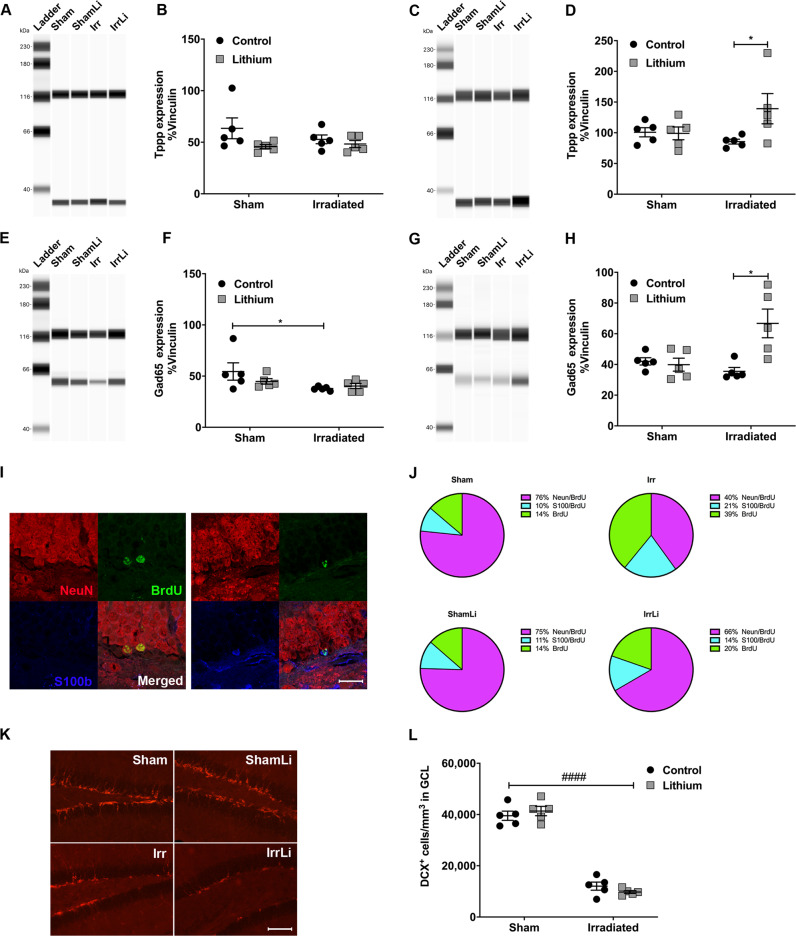
Lithium regulated the expression of cell cycle and neuronal transmission proteins in vivo and reverted the irradiation-induced changes in NSPC fate progression in the mouse DG. **a** Representative western blot lanes of Tppp migration at PND 77. Tppp signal was observed at the MW of 37 kDa. Vinculin, used as a loading control, was observed at the expected MW of 118 kDa. **b** Dot plot graph showing the quantification of the normalized chemiluminescence peak of Tppp against its loading control vinculin at PND 77. Tppp expression levels in the DG were unaltered in all treatment groups. Two-way ANOVA: irradiation (*F*_1,16_ = 0.4998, *p* = 0.4898), lithium (*F*_1,16_ = 3.654, *p* = 0.0740). **c** Representative western blot lanes of Tppp migration at PND 91. **d** Dot plot graph showing the quantification of the normalized chemiluminescence peak of Tppp against its loading control vinculin at PND 91. The Tppp expression level in the DG was significantly increased in the IrrLi group (*p = 0.0313). Two-way ANOVA: irradiation (*F*_1,16_ = 0.7927, *p* = 0.3865), lithium (*F*_1,16_ = 3.414, *p* = 0.0832). **e** Representative western blot lanes of GAD65 migration at PND 77. GAD65 signal was observed at the MW of 65 kDa. Vinculin, used as a loading control, was observed at the expected MW of 118 kDa. **f** Dot plot graph showing the quantification of the normalized chemiluminescence peak of GAD65 against its loading control, vinculin at PND 77. GAD65 expression level in the DG was significantly reduced in the Irr group (*p = 0.0431) but no change was observed in the other groups. Two-way ANOVA: irradiation (*F*_1,16_ = 5.232, *p = 0.0361), lithium (*F*_1,16_ = 0.599, *p* = 0.4502). **g** Representative western blot lanes of GAD65 migration at PND 91. **h** Dot plot graph showing the quantification of the normalized chemiluminescence peak of GAD65 against its loading control vinculin at PND 91. GAD65 expression level in the DG was significantly increased in the IrrLi group (**p = 0.0017). Two-way ANOVA: irradiation (*F*_1,16_ = 3.543, *p* = 0.0781), lithium (*F*_1,16_ = 7.228, *p = 0.0161. **i** Representative confocal images of NeuN (red), BrdU (green), and S100β (blue) immunoreactivity in the GCL at PND 105 depicting colocalization of BrdU and NeuN in mature neurons (upper panel) and colocalization of BrdU and S100β in an astrocyte (lower panel). Scale bar = 20 µm. ×63 magnification. **j** Pie chart of the percentages of BrdU cells double labeled for NeuN or S100β. The percentages of NeuN^+^/BrdU^+^ cells were significantly increased in the IrrLi (**p = 0.0031) but not in ShamLi (*p* > 0.9999) group. Two-way ANOVA: irradiation (*F*_1,14_ = 22.5, ***p = 0.0003), lithium (*F*_1,14_ = 7.02, *p = 0.0191). The percentages of S100β^+^/BrdU^+^ were significantly increased after irradiation but partly normalized by lithium treatment. Two-way ANOVA: irradiation (*F*_1,14_ = 4.89, *p = 0.0442), lithium (*F*_1,14_ = 0.831, *p* = 0.3774). The remaining percentages of BrdU^+^ cells were significantly increased in the Irr group (*p = 0.0137) but not altered in the other groups. Two-way ANOVA: irradiation (*F*_1,14_ = 13.1, **p = 0.0028), lithium (*F*_1,14_ = 5.12, *p = 0.0401). **k** DCX immunoreactivity in the GCL at PND 105 in different treatment groups. Scale bar = 100 µm. ×20 magnification. **l** Interleaved dot plot graph of the quantification of DCX^+^ cells in the GCL showing the effect of irradiation (****p < 0.0001) on the density of DCX-positive cells in the GCL. Two-way ANOVA: irradiation (*F*_1,16_ = 372.2, ^####^*p* < 0.0001), lithium (*F*_1,16_ = 0.02213, *p* = 0.8836). Error bars indicate SEM. ***p < 0.001; **p < 0.01; *p < 0.05

To be able to validate the DNA methylation and RNA sequencing data in our in vivo model, we investigated the protein expression levels of Tppp and GAD65 in hippocampal tissue by immunoblotting. The levels of Tppp on PND 77 were the same in all treatment groups (Fig. 4a, b and Supplementary Fig. 1A), whereas at PND 91 a significant increase in Tppp expression was observed in the irradiation lithium-treated group (Fig. 4c, d and Supplementary Fig. 1B). The levels of GAD65 on PND 77 were significantly reduced in the irradiation group but unaltered in the other groups (Fig. 4e, f and Supplementary Fig. 1C), however on PND 91 its expression increased significantly in the irradiation group treated with lithium (Fig. 4g, h and Supplementary Fig. 1D).

### Lithium prevented the irradiation-induced changes in nspc fate progression observed 4 weeks after its discontinuation

To assess the survival and differentiation of NSPCs at a later time in the neurogenic process, we conducted a triple immunostaining of BrdU, NeuN and S100β in the DG on PND 105 (Fig. 4i). We found that irradiation drastically reduced the proportion of NeuN/BrdU^+^ cells (from 76 to 40%) and increased the proportion of S100β/BrdU^+^ cells (from 10 to 21%) (Fig. 4j). Lithium after irradiation was able to restore the percentage of neuronal commitment from 40 to 66%, and astrocyte commitment from 21 to 14%, approaching sham levels. Lithium did not change the cell fate in sham animals. In addition, irradiation significantly increased the percentage of surviving cells (BrdU^+^) that were neither of neuronal, nor astrocytic lineage. To further assess whether lithium had a long-lasting effect on the positive modulation on DCX^+^ cells, we analyzed the density of the DCX^+^ cells in the GCL on PND 105 (Fig. 4k). The density was still significantly decreased after irradiation, consistent with previous models (Boström et al. 2013) (Fig. 4l). In addition, there was no significant difference in the density of DCX^+^ cells between the vehicle and the lithium-treated mice, neither in the irradiated nor in the sham groups (Fig. 4l). In addition, quantification of BrdU^+^ density (cells/mm^3^ in GCL) at PND 105 showed that survival was decreased by irradiation while lithium increased the survival of adult-generated DG cells in the sham group (Sham = 8064 vs ShamLi = 10,791, **p = 0.0016) but not in the irradiated group (Irr = 3459 vs IrrLi = 4469). Together, this indicates that lithium treatment promotes proliferation of NSPCs and that discontinuation of the treatment promotes a wave of neuronal differentiation, and at the same time prevents the wave of irradiation-induced astrocytic differentiation.

### Lithium ameliorated irradiation-induced spatial learning and memory retention deficits 4 weeks after its discontinuation

**Fig. 5:**
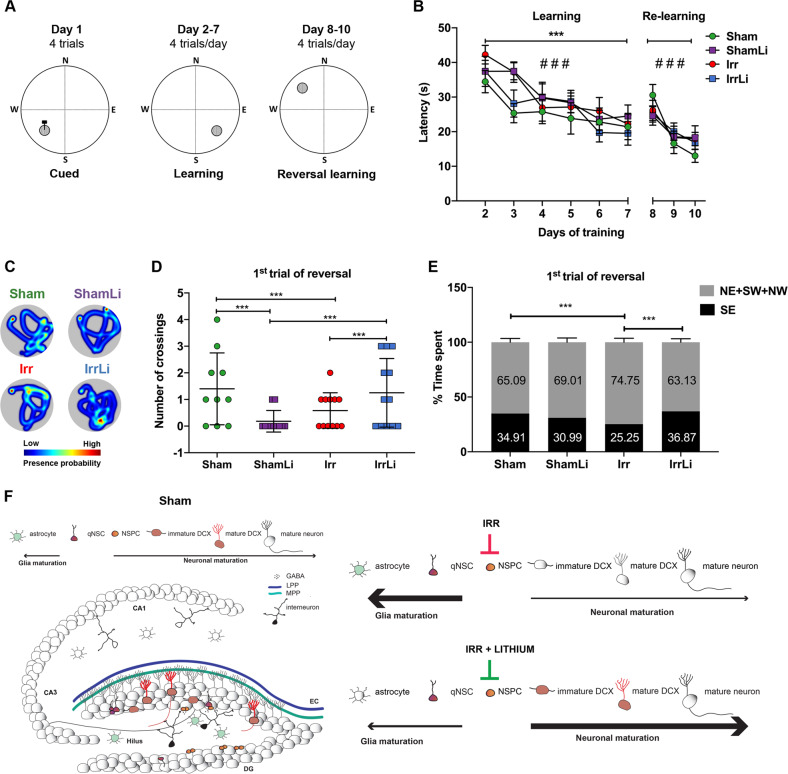
Lithium rescued the irradiation-induced deficits on hippocampal-dependent learning and memory. **a** Experimental protocol of the Morris water maze task; the platform was moved between the cued, learning, and reversal learning training sessions. **b** All groups successfully learned to navigate to the hidden goal over time in both learning (GEE for latency: *W*_5,270_ = 64.960, ^###^*p* < 0.001) and reversal learning test (GEE for latency: *W*_2,135_ = 37.8941, ^###^*p* < 0.001), but the Sham and IrrLi mice found the hidden platform faster than the Irr and ShamLi mice during the first acquisition period (days 2–7) (GEE group effect: *W*_3,270_ = 29.384, ***p < 0.001; post hoc tests: Sham vs. ShamLi *p* = 0.004, Sham vs. Irr *p* < 0.001, IrrLi vs. ShamLi *p* = 0.005, IrrLi vs. Irr *p* < 0.001). **c** Representative heat maps for all groups of animals of the first trial of the reversal learning. **d** Number of crossings over the previous goal position. The IrrLi and Sham mice showed higher preference for the previous position of the platform compared with the Irr and ShamLi mice (GLM group effect: *W*_3,45_ = 58.844, *p* < 0.001; post hoc tests: Sham vs. ShamLi ***p < 0.001, Sham vs. Irr ***p < 0.001, ShamLi vs. IrrLi ***p < 0.001, Irr vs. IrrLi ***p = 0.001). **e** Time spent in the quadrants during the first trial of the first day of reversal learning. There was a significant group effect (GLM: *W*_3,45_ = 23.068, *p* < 0.001). Sham and IrrLi animals exhibited a stronger preference than the Irr mice for the quadrant where the platform was located during the acquisition period (post hoc tests: Sham vs. Irr ***p < 0.001, Irr vs. IrrLi ***p = 0.001), while ShamLi had an intermediate value compared with the three other groups. **f** Schematic drawing of the hippocampal network on the left. Input signals from the entorhinal cortex (EC) are carried through two connectional routes made of the axons of the medial (light green) and lateral (blue) perforant pathways, MPP and LPP respectively. These axons establish stable synapses with the dendrites of the mature granule cell neurons (gray) and weak ones with the immature doublecortin (DCX) cells (red) in the granule cell layer (GCL). The subgranular zone (SGZ) is located at the boundary of the GCL and the hilus, where quiescent neural stem cells (qNSC) give rise to amplifying neural progenitors (ANP) allowing the continuous neuronal re-population of the dentate gyrus (DG). The qNSC and ANPs are multipotent stem cells, capable of giving rise to astrocytes, oligodendrocytes and neurons. The input signal from the DG is relayed to the proximal Cornus Ammonis region (CA3) through the axons of the mature granule cells that form the mossy fiber projection. The signal transduction continues to the CA1 region through the Schaffer collateral fibers and ultimately to further cortical areas. Parvalbumin (PV) interneurons in the hilus are important in modulating, through feedback and feedforward inhibition, the input signals and NSC proliferation and integration through the release of the neurotransmitter gamma-aminobutyric acid (GABA). Right top: a schematic representation of the effects of irradiation on DG. The number of astrocytes is increased while the number of ANPs is decreased. The neuronal differentiation process is decreased in favor of an astrocytic fate progression. Right bottom: a schematic representation of the effects of lithium on the irradiated DG. Lithium acts by increasing ANPs cell number and promoting neuronal fate progression as compared with astrocytic differentiation

Mice were assessed for spatial learning, memory, and reversal learning in the Morris water maze (MWM), a well-established hippocampal-dependent task (Fig. 5a) [45]. No visual deficits were observed during the cued version of the MWM performed the first day, as judged by the latency to find the visible platform (*p* > 0.05, data not shown), so all mice were included in the subsequent phases of the task. During the first acquisition period (days 2–7), all groups showed learning, since the mean latency and distance swum to find the hidden platform decreased over time (Fig. 5b and Supplementary Fig. 2). There was a statistically significant group effect, but no group × day interaction and post hoc comparisons revealed that the Sham and IrrLi mice showed better learning than the ShamLi and Irr mice, i.e., shorter latency and distance moved to find the hidden platform over the training days. During the reversal learning all groups were able to learn the new position of the platform, as judged by reduced latency and distance swum between days 8 and 10 of the task (Fig. 5b and Supplementary Fig. 2). No group effect or group × day interaction was observed for the reversal learning. Importantly, all mice displayed normal and similar swim speeds throughout the task (days 1–10) (Sham 19.51 ± 0.28 cm/s, ShamLi 19.62 ± 0.36 cm/s, Irr 19.82 ± 0.43 cm/s, IrrLi 19.89 ± 0.28 cm/s) and had no difficulties climbing onto the submerged escape platform.

As evident in the heat maps (Fig. 5c), in the first trial of the first day of reversal learning, which can be considered a memory retention test, there were significant group differences in the number of crossings over the previous position of the platform (Fig. 5d), as well as in the time spent in the quadrant where the platform was placed during the previous days of training (Fig. 5e). The number of goal crossings was higher for the Sham and IrrLi mice compared with the ShamLi and Irr mice, indicating better memory retention in the Sham and IrrLi groups. In addition, Sham and IrrLi animals exhibited a stronger preference than the Irr mice for the quadrant where the platform was located during the acquisition period, while ShamLi had an intermediate value compared with the three other groups.

## Discussion

### Lithium is effective even when introduced long after the injury

Cranial radiation therapy is a major cause of long-term complications in pediatric patients [1]. Here we chose to investigate female subjects due to their propensity to suffer more from postirradiation cognitive deficits [6, 46, 47], and given that lithium effects are not dependent on sex we argued that a successful lithium treatment in females is likely to aid male subjects to the same extent [48, 49]. These complications include late-occurring cognitive impairments, and a negative impact on social competence [3, 50]. It has been demonstrated in animal models that preserving or promoting neurogenesis helps attenuate the cognitive deficits observed in irradiated mice and rats [26, 40]. Progress has been made in identifying mechanisms underlying the neuroprotective effects of lithium in rodent models of brain injury, including antiapoptotic effects [23, 25, 51]. Regenerative effects were demonstrated when lithium was shown to reduce brain tissue loss by 39% after HI in the immature brain even when it was administered 5 days after the injury [21] in a model where neuronal cell death peaks 1–2 days after HI, but the underlying mechanisms have not been characterized. Delayed administration of lithium towards preservation of neurogenesis following whole-brain cranial irradiation has never been reported. In this study we demonstrate that lithium treatment in female mice reverses irradiation-induced loss of hippocampal neurogenesis and cognitive impairment even when introduced long after the injury.

Following irradiation-induced hippocampal injury, the neurogenesis cascade within the GCL undergoes several long-lasting changes: an increase in NSPC apoptosis, a protracted decrease in NSPC proliferation, a decreased propensity for differentiation of those NSPCs, where gliogenesis is favored over neurogenesis, and onset of inflammation [10, 25, 52]. We now demonstrated that on PND 77, after 4 weeks of continuous lithium treatment of irradiated mice, lithium promoted proliferation of NSPCs, as indicated by the increased density of BrdU^+^ cells in the GCL. In a separate study, we found that lithium increased the proliferation of mouse hippocampal-derived NSPCs in vitro, and drove them much faster through the G1 phase of the cell cycle compared with control NSPCs [41]. The cells in the dentate gyrus responding to pro-neurogenic stimuli, such as physical exercise, enriched environment, and antidepressants, are primarily amplifying neural progenitors [53–56]. Hence, it is likely that lithium increases the rate of symmetric divisions of the amplifying neural progenitor population, which would be therapeutically beneficial since those cells represent a renewable source of neuronal precursors.

Why would delayed onset of lithium treatment be selected? The effects of lithium on tumor cells are only partly explored [26], there is a potential risk that lithium may enhance not only neurogenesis in normal brain tissue, but also the proliferation of remaining tumor cells. For this reason, “pharmacological rehabilitation” of cognitive late effects after brain tumor treatment may be safer to perform a couple of years after the end of treatment, when the risk of tumor relapse is lower. Some of the pro-proliferative effects of lithium are based on pathways which have been well described [29] but still not fully understood. Certain effects of lithium on brain tumor cells or leukemia cells have been described: while lithium might not protect tumor cells [24, 26], or might even act as an antitumor agent in certain types of medulloblastoma [36, 37], glioblastoma [39], glioma [57] or leukemia [58], its action on other tumor cell types remains uncertain. Therefore, it may be advisable to wait until the anticancer therapy is finished. Thus, to show, as we did, that lithium can rescue neurogenesis long after irradiation, is an important step before validating its use in childhood brain tumor survivors. Lithium treatment could then be used for all survivors who have already been treated and suffer from late-appearing cognitive deficits.

### Intermittent treatment is important

From a conceptual point of view, it is crucial to investigate if it is at all feasible to enhance neurogenesis and cognitive function even long after radiotherapy. We showed for the first time in vivo that discontinuation of lithium could restore neuronal fate progression in the irradiated brain, thereby counteracting the irradiation-induced astrocytic differentiation of NSPCs previously described [59, 60], further suggesting a role for lithium in restoring neurogenesis after cranial radiotherapy. Continuous oral lithium administration with stable serum concentrations promoted NSPC proliferation but prevented neuronal differentiation. Discontinuation allowed differentiation and integration to occur over the following 4 weeks. Hence, we surmise that a sequential therapeutic regimen, for example 1 month with lithium and 1 month without it, would be more effective in the treatment of cognitive late effects. Another advantage would be that the side effects frequently reported in patients treated with lithium [61] could be better tolerated. This is a novel concept that deserves to be tested in patients.

### Identification of mechanisms

Studying both rat neural stem cells in vitro and mouse hippocampal neurogenesis in vivo enabled us to identify molecular mechanisms underlying the positive effects of intermittent lithium treatment. Two indices related to the functional integration of DCX^+^ cells into the GCL are the orientation of integrating DCX^+^ cells, and the maturity of their dendritic processes [62, 63]. Type-3 neuroblasts possess an elongated cell body, flanked by processes that lie tangential to the SGZ, suggestive of an early stage of maturation, while newborn neurons have a radial process to the SGZ that is indicative of functional integration into the granule cell layer [64]. By phenotyping the orientation of dendritic processes in cells double labeled with BrdU and DCX, we found, as previously reported [40, 65], that irradiation decreased radial migration, and increased the number of parallel dendritic processes in a different subset of neurons within that population of cells. In irradiated mice treated with lithium, the percentage of cells with parallel dendrites was reduced but the radial migratory pattern was unaffected. Arguably, lithium protects against irradiation damage by limiting the number of cells with parallel dendritic processes so that immature neurons are not maintained in that stage. Overall, these data suggest that lithium discontinuation is pivotal for the late critical period of newborn cell survival as well as their structural and synaptic integration. Here irradiation persistently reduced dendritic complexity (number, length, and area of branches) similar to previous studies in which these changes correlated with altered expression of molecular indices of synaptic plasticity as well as impaired behaviorally functional measures [66, 67]. We extended these findings and showed that continuous lithium treatment followed by a period without lithium acts positively on dendritic maturation and complexity, which is a morphometric parameter that has important functional implications in memory function and anti-stress mechanisms [68]. We hypothesized that this is attributable to key regulatory proteins involved in cytoskeletal rearrangements (Tppp) and synaptic transmission (GAD65). Tppp expression is known to increase the stability of microtubule networks, thereby playing a crucial role in cell differentiation [69], while the knockdown of GAD65 impairs the maturation of newborn granule cells [70]. Strikingly, both Tppp and GAD65 were upregulated at the mRNA level in vitro, and at the protein level in vivo, 2 weeks after discontinuation of lithium (PND 91), further confirming the positive modulatory effects of lithium on the neurogenic process as well as the importance of discontinuing the proliferative drive of lithium to allow integration of the newborn cells. The decrease in GAD65 expression after irradiation is in accordance with previous observations demonstrating that irradiation decreases the number and activation of GABAergic, parvalbumin-positive cells in the infralimbic cortex [71], while others reported increased GABA release in cannabinoid1 interneurons, decreased tonic GABAergic signaling, and increased principal cell activation in the hippocampus [72]. Both types of interneurons modulate principal cells activation and we previously demonstrated that early life irradiation increases excitability and impairs long-term potentiation in the adult dentate gyrus [73]. One hypothesis is that irradiation impinging on the inhibitory network disrupts long-term the synchronous activity of different brain regions and it is possible that lithium by increasing the expression of GAD65 in the irradiated brain normalizes the overall inhibition-excitation balance. In addition, we found that the increased expression of both Tppp and GAD65 was accompanied by a decrease in methylation of these two regulatory genes in the irradiated group treated with lithium, indicating that the positive effects of lithium after irradiation are attributed to an epigenetic regulation of genes important for cell fate, maturation, and integration.

It is noteworthy that the lithium-induced upregulation of Tppp and GAD65 occurred only in the irradiated, injured tissue. This demonstrates that intermittent lithium treatment has unidentified targets in the irradiated brain.

More so, when adult-born DG cells undergoing maturation were transiently reduced, this proved detrimental for learning and memory [74], as this stage of maturation (4–8 weeks) has a critical role in these functions [75]. Herein, we show that when the pro-proliferative effect of lithium on neurogenesis ends at 4 weeks after the cessation of lithium treatment, memory retention in the MWM is improved on the first day of reversal in the IrrLi group compared with the Irr group. We show that the stage of maturation of DG cells seems critical for learning and memory, and the long-term functional effects of irradiation on adult-born DG cells are obtained by discontinuing lithium treatment, as summarized by Fig. 5f. It remains to be determined why the ShamLi group performed poorly and similarly to the Irr group. One possible explanation is that despite increased survival of BrdU^+^ cells in the ShamLi group, these cells may have impaired maturation. We did find that lithium treatment per se reduced dendritic branching (number of intersections) in the ShamLi, but not in the IrrLi group. Although molecular parameters investigated here do not point to such impairment, we speculate that aberrant neurogenesis may contribute to these deficits as reported in similar studies [76–78]. More in-depth analysis of their function is warranted. On the other hand, it is striking how lithium treatment rescues neurogenesis and cognition, suggesting that an injured brain thus is far more responsive to lithium than an intact brain that has an unchallenged microenvironment.

Lithium discontinuation in bipolar patients may attenuate the therapeutic response upon re-treatment [79], but discontinuation is nevertheless advised in patients that have higher tendencies to develop side effects [80]. While repeated lithium discontinuation and re-treatment may reduce side effects, it remains to be shown that rescued cognition can be maintained. In summary, lithium has the potential to become the first pharmacological treatment of cognitive late effects in childhood cancer survivors. The optimal dosing regimen remains to be determined but we hypothesize that an intermittent lithium treatment would work.

## Materials and methods

### Animals and ethical permissions

C57BL/6 female mice were obtained from Charles River Laboratories (Sulzfeld, Germany). Pups were delivered with their respective dams and were separated at weaning (PND21). Mice were kept under standard temperature, humidity, and daylight conditions (12-h light:dark cycle) and were provided with food and water *ad libitum*. All experiments were conducted in accordance with the national and European laws for the use of animals in research (EU Directive 2010/63/EU) and were approved by the local ethical committee (Ethics Committee on Animal Research, Stockholm North). The ethical identification numbers were: N9–12, N248–13 and N163–15 for the in vivo study, and N284–11 and N190114 for the in vitro study.

### Irradiation procedure

Mice were anaesthetized using isoflurane at 4% for induction followed by 1.5–2% throughout the procedure. Mice were placed on a custom-made Styrofoam frame in prone position (head to gantry), and the frame placed inside an X-ray system (Precision X-RAD 320, North Branford, CT, USA) setup in-house for in vivo targeted radiotherapy research with an energy of 320KV, 12.5 mA and a dose rate of 0.75 Gy/min. The whole brain was irradiated with a radiation field of 2 × 2 cm. A single dose of 4 Gy was delivered to each animal on postnatal day (PND) 21. The source-to-skin distance was approximately 50 cm. The sham-irradiated mice were anesthetized but not irradiated.

### Lithium in vivo administration

Female littermates (4–6 animals in each cage) were randomly assigned to lithium chow (2.4 g/kg Li_2_Co_3_, 0.24%, TD.05357 Lithium Carbonate Diet 2018, Harlan laboratories, Netherlands) or control chow diet (T.2918.CS, Harlan laboratories, Venray, The Netherlands). This regimen was determined in our previous study [81] and was sufficient to yield a lithium serum concentration of 0.7–0.9 mM in mice, which is equivalent to the commonly used 0.6–1.2 mM therapeutic range in humans. The lithium chow was maintained for 4 weeks, from PND 49 to PND 77 (Fig. 1a in vivo study design).

### Immunohistochemistry

Animals were injected intraperitoneally with 50 mg/kg BrdU (5-Bromo-2′-Deoxyuridine) (B5002, Sigma-Aldrich, St. Louis, MO, USA) for 5 days starting from PND 72. Animals were sacrificed for analysis at three different time points, each separated 2 weeks apart: PND 77, PND 91, and PND 105.

Immunohistochemistry and quantification were performed as previously described [81]. The following primary antibodies were used: rat anti-BrdU (1:500, AbD Serotec, Kidlington, UK) and goat anti-DCX (1:100, Santa Cruz Biotechnology Inc., CA, USA). The following secondary antibodies were used: biotinylated donkey anti-goat IgG (H + L) (Molecular Probes, Paisley, UK) and biotinylated donkey anti-rat IgG (H + L) (Jackson ImmunoResearch Europe, Suffolk, UK). For immunofluorescence, the following primary antibodies were used: rat monoclonal anti-BrdU (1:500, AbD Serotec, Kidlington, UK), goat anti-DCX (1:200, Santa Cruz Biotechnology Inc., Dallas, TX, USA), mouse monoclonal anti-NeuN (neuronal nuclei) (1:200, Merck Millipore, Billerica, EMD Millipore Corporation, Temecula, CA, USA) and rabbit polyclonal anti-S100β calcium binding protein Abcam, Cambridge, UK). The following secondary antibodies were used: Alexa Fluor^®^ 555 donkey anti-mouse IgG (H + L), Alexa Fluor^®^ 488 donkey anti-rat IgG (H + L) (Invitrogen, Life technologies, Carlsbad, CA, USA), Alexa Fluor^®^ 555 donkey anti-goat IgG (H + L) (Biotium, Hayward, CA, USA), and Alexa Fluor^®^ 633 donkey anti-rabbit IgG (H + L) (Biotium, Hayward, CA, USA). Sections were mounted in ProLong^®^ Gold Antifade Reagent with DAPI (#8961, Cell Signaling Technology, Danvers, MA, USA). For DCX^+^ cell analysis and density measurements, a fluorescent microscope was used and the total number of cells and contour areas were estimated using unbiased counting software (Stereo Investigator, MicroBrightField Inc.; Colchester, VT, USA). The other fluorescent analyses were conducted using a confocal microscope as previously reported (van Praag, Kempermann, and Gage 1999) (Axio Observer-Z1 with ZEN lite software, Carl Zeiss AG, Oberkochen, Germany). Only cells with an entire, clearly visible cell body were counted.

### Dendrite reconstruction and morphometric analyses

Imaging of dendritic arbors on PND 77 and PND 91 was performed using a confocal microscope by acquiring images with 1 µm intervals using a 20X objective lens (X20/0.8 Plan-Apochromat lens, Carl Zeiss) (1024 × 1024 pixels). The entire dendrites and cell bodies of each neuron in the captured images were traced manually using Neurolucida^®^ software (MBF Bioscience, Williston, VT, USA). Three-dimensional analysis of the reconstructed neurons was performed and the total dendritic length, dendritic complexity and the cell body area were measured using Neurolucida Explorer^®^ software (MBF Bioscience). A branch order was assigned to each dendrite and then the dendritic complexity was calculated as follows; dendritic complexity = [Sum of the terminal orders + Number of terminals] × [Total dendritic length/Number of primary dendrites] (Pillai et al. 2012). This analysis was performed after DCX staining of three animals (1:12 series) randomly and blindly chosen in each group; 15–20 cells per animal were randomly chosen by an investigator blinded to the treatment of the animals. To measure the extent of dendritic arborization away from the soma at different distances, Sholl analysis (Sholl 1953) was conducted by counting the number of dendritic intersections for a series of concentric spheres at 10-µm intervals. The center of a concentric sphere was placed at the centroid of the soma. Immature neurons which satisfied the following criteria were selected for analysis; (i) fully labeled DCX^+^ cells at a postmitotic stage [63], (ii) neurons were relatively isolated from neighboring DCX-positive neurons to avoid interfering with analysis, (iii) the soma located in the SGZ or within the inner one-third of granular cell layer of the upper blade of the dentate gyrus, to compare the neurons which are at the same stage of immaturity. Truncated cells were excluded from the analysis. To assure impartiality morphometric analysis was performed blindly by the same investigator.

### Protein quantification

Hippocampal tissue was processed for protein quantification as previously described [82]. The Wes capillary electrophoresis system (Protein Simple-Bio Techne, San Jose, CA) was used for all protein quantitation. Sample aliquots were thawed and diluted to 0.2 μg/μl for all targets using 0.1× Sample Buffer and 5× Master Mix (1:1 mix of 400 mM DTT and 10× Sample Buffer) according to manufacturer’s instructions. Samples were denatured at 95 °C for 5 min. Rabbit α-GAD65 (PA5-22260, Thermo Fisher Scientific, USA) and Rabbit α-Tppp (ab92305, Abcam, USA) were used at a concentration of 1:200 and 1:2000, respectively. Rabbit α-Vinculin was used as housekeeping control at a concentration of 1:200,000. An α-rabbit secondary antibody was provided in the kit and was used according to manufacturer’s instructions. For protein quantification the total chemiluminescent peak area was normalized to the respective reference capillary of the housekeeping control. The normalized peak area under the curve was used for protein quantification.

### Embryonic telencephalic NSPC culture and LiCl exposure procedures

Primary cultures of NSPCs were established as previously described [83, 84]. Cells were obtained from embryonic telencephalon (*n* = 10–12/cell preparation) dissected in HBSS (Life Technologies, Carlsbad, CA, USA) from timed pregnant Sprague Dawley rats (Harlan Laboratories, Harlan, The Netherlands), (Ethical Permit: N284/11 and N190114) at E15.5 (the day of copulatory plug defined as E0). The tissue was mechanically disrupted, and meninges and larger cell clumps were allowed to sediment for 10 min. The cells were plated at a density of 40,000/cm^2^ on dishes precoated with poly-l-ornithine and fibronectin (both from Sigma-Aldrich, St. Louis, MO, USA; Stockholm, Sweden). Cells were kept in enriched N-2 medium with 10 ng/ml of basic fibroblast growth factor (R&D systems, Minneapolis, MN, USA) added every 24 h and medium changed every alternate day to keep the cells in an undifferentiated and proliferative state. Cells were passaged every 5 days by detaching through scraping in HBSS. Thereafter, the cells were gently mixed in N-2 medium and plated at 1:4 density. To investigate LiCl (Sigma-Aldrich, St. Louis, USA) effects, we exposed passage 3 (P3) NSPCs from 12 h before irradiation to LiCl (3 mM), as previously described [41]. A photon ^60^Co irradiation source was used to expose the NSPCs at a set distance of 80 cm and an absorbed dose of 2.5 Gy. P3 cells were harvested 24 h after irradiation for gene expression analysis (Fig. 3a, in vitro study design).

### RNA, cDNA, and RT-qPCR

For real-time qPCR, total RNA from culture NSPCs was extracted using RNeasy Mini Kit (Qiagen) and stored at −80 °C until further use. Integrity and concentration of extracted RNA were measured using Qubit (Thermo Fisher Scientific). cDNA was synthesized from extracted RNA using High Capacity cDNA Reverse Transcription Kit (Thermo Fisher) according to the manufacturer’s protocols. Quantitative real-time PCR was performed with Platinum SYBR Green qPCR Supermix-UDG (Thermo Fisher Scientific) together with site-specific primers. Expression levels were normalized to housekeeping gene, TATA-box binding protein levels.

### RNA sequencing

Illumina TruSeq Stranded mRNA sample preparation kit with 96 dual indexes (Illumina, CA, USA) was used to prepare RNA libraries for sequencing, respectively four biological samples per condition (Sham, ShamLi, Irr, IrrLi), for a total of 16 samples. The protocols were automated using an Agilent NGS workstation (Agilent, CA, USA) using purification steps as described previously [85, 86]. Quality control was checked with 2100 Bioanalyzer (Agilent) with all 16 samples having RIN values of 8 or above. Libraries were sequenced on HiSeq 2500 (HiSeq Control Software 2.2.58/RTA 1.18.64) with a 2 × 126 setup using HiSeq SBS Kit v4 chemistry to an average depth 32.3 M reads (30.4–34.5). The data were briefly processed as following: FastQC/0.11.5 quality check was performed on raw sequencing reads, Star/2.5.1b was used to align the reads to the reference genome Rattus norvegicus genome Rnor_6.0 and QualiMap/2.2 was used to evaluate the quality of this alignment. Reads overlapping fragments in the exon regions were counted with featureCounts (subread/1.5.1) using default parameters, i.e., fragments overlapping with more than one feature and multi-mapping reads were not counted.

Differential expression analyses were performed under R/3.3.3 using EdgeR/3.16.5 package. Low count reads were filtered by keeping reads with at least 1 read per million in at least two samples. Counts were normalized for the RNA composition by finding a set of scaling factors for the library sizes that minimize the log-fold changes between the samples for most genes, using a trimmed mean of M values (TMM) between each pair of samples. Design matrix was defined based on the experimental design, genes-wise glms models were fitted and likelihood ratio tests were run for the selected group comparisons. RNA-seq data have been deposited in the ArrayExpress database at EMBL-EBI (www.ebi.ac.uk/arrayexpress) under accession number E-MTAB-7238.

### MeDIP-qPCR

MeDIP-qPCR was performed using MagMeDIP kit (Diagenode) according to the manufacturer’s instructions. Briefly, rat NSPCs cells were lysed and the DNA was extracted using phenol:chloroform:isoamyl alcohol (25:24:1) (Sigma-Aldrich), purified using Purelink Genomic DNA kits (Invitrogen, now Thermo Fisher), fragmented using Bioruptur (Diagenode), and immunoprecipitated with the antibody anti-5′-methylcytosine (Diagenode), following MagMeDIP kit settings. DNA concentration was measured using Qubit dsDNA HS Assay Kit (Thermo Fisher). Immunoprecipitated DNA was quantified using RT-qPCR, as described above, and the temperature profile used was: 95 °C for 7 min, 40 cycles of 95 °C for 15 s, and 60 °C for 1 min, followed by 1 min 95 °C. Tppp, Gad2 promoter primers (Qiagen) and Methylated DNA and unmethylated DNA control primers (Diagenode) were used as internal controls (Supplementary Fig. 3). The efficiency of methyl DNA immunoprecipitation was expressed as a relative to the percentage of the input DNA using the following equation:

$$	\% \left( {meDNA \,-\, IP \div Total\;input} \right) \\ 	 =\, 2 \wedge \left[ {\left( {Ct\left( {10\% input} \right) \,-\, 3.32} \right) \,-\, Ct\left( {meDNA \,-\, IP} \right)} \right] \times 100\%$$

### Behavioral assessment

Three different cohorts (*n* = 15–16/cohort) of mice were used for the behavioral study. From each litter employed, animals were randomly assigned to all four experimental groups. Animals were group-housed (*n* = 3–4 mice/cage) and in each cage animals from different experimental groups were included. All animals were naïve to the tasks. Testing took place between 10.00 and 16.00 under low illumination (100–150 Lux) to reduce stress but strong enough to provide proper visibility of environmental cues. On the days of testing, animals were brought in their home cages to the testing room and allowed to rest and habituate for at least 1 h before the beginning of the experiment. The estrous phase was determined, based on vaginal smears, on the last day of testing to avoid additional stress to the animals. We verified that all groups of animals showed all stages of the estrous cycle without any apparent differences in their relative frequencies (data not shown).

### Morris water maze

The MWM was performed as previously described with slight modifications [87–90]. Mice were trained in the reference and reversal memory versions of the MWM to locate a hidden escape platform (10 cm × 10 cm) in a circular pool (120 cm diameter). The pool was placed in a room with stable temperature and humidity. The platform was submerged 1 cm under the water surface and its position was fixed relative to extra-maze 3D visual cues (O’Hara & Co. ltd, Tokyo, Japan). Water was made opaque with non-toxic white dye (Opacifier for MWM, cat. number OP301, Viewpoint, Civrieux, France) and kept at a temperature of 22 ± 1 °C. On the first day, the cued version of the MWM took place, during which each mouse was allowed four trials to locate the platform guided by a credit card size plastic “flag”. The reference version of the MWM followed for 6 consecutive days. The platform was moved 90° counterclockwise from the position used during the cued test (Fig. 5a). Each session (day) consisted of four trials, separated by 15-min intervals, each trial having a maximum duration of 60 s. In each trial the starting position was different, in a pseudorandom order, and each day included entry points in all four quadrants of the pool. Each trial ended when either the animal found the platform or after 60 s, in which case the animal was guided to the platform by the experimenter. In either case, it remained on the platform for 20 s and was then returned to its home cage. On day 8, a reversal version of the MWM followed for 3 consecutive days. The platform was moved 180°, to the quadrant opposite to the one used during reference memory training, and the animals entered the water maze again from four different entry points per day in a pseudorandom order. The behavior of the animals was recorded and analyzed using the Noldus Ethovision software (Ethovision XT 14.0, Noldus Information Technologies, Wageningen, Netherlands). For each trial, for each mouse, the latency (time in sec to climb onto the platform) and the distance swam (total distance in cm to climb on the platform) were determined and an average was calculated for each day. For the first trial of the reversal learning, we calculated the % of time spent in the quadrant where the platform was during the learning phase, and the number of crossings over the “previous position” of the platform. In order to exclude the possibility that any learning or memory deficits observed in the MWM were due to deficits in motor activity, swim speed (cm/sec) was calculated.

### Statistical analysis

Statistical differences in immunohistochemistry, protein quantitation, and gene expression analysis were calculated using a two-way ANOVA analysis followed by a Bonferroni post hoc test for multiple comparisons correction using GraphPad Prism^®^ (La Jolla, CA, USA). For methylation analysis, Kruskal–Wallis test for multiple comparisons, followed by Dunn´s multiple comparisons test was performed. For dendritic analysis, linear mixed models with a random intercept for each animal were used to account for within-individual dependencies, using R version 3.3.3 (The R Foundation for Statistical Computing, Vienna, Austria). As data were not normally distributed, natural logarithmic transformation was used to satisfy the requirement for normality. To ensure normal distributions, variables were plotted as Q–Q plots using SPSS (IBM SPSS Statistics, version 21.0, Chicago, IL, USA). For Sholl analysis, two-way ANOVA followed by post hoc Bonferroni test was performed using GraphPad Prism (version 7.03, GraphPad Software, La Jolla, CA, USA). All quantifications were done in a blinded fashion. The analysis of behavior was performed using SPSS v. 24 software (SPSS Inc., Chicago, IL, USA) by an investigator blinded to the group identity of the animals. Any group effects on the latency and distance to reach the platform, as well as on the velocity during the learning and reversal learning in the MWM, were assessed using the generalized estimated equations model. The animal ID was used as a subject variable, the day as a within-subject variable, the group, day and group × day as predictor factors, and the group (litter) as build nested predictor factor. Any group effects on latency and distance moved during cued learning as well as on the % time spent in the quadrant where the platform was during learning, and on the number of crossings over the old position of the platform during the first trial of the reversal learning, were assessed using the generalized linear model, with the group as predictor factor and the group (litter) as a build nested predictor factor. In the case of statistically significant effects, Bonferroni post hoc tests were used to determine specific group differences. In all analyses, the data represent the mean ± SEM. The level of significance was set at *p* < 0.05.

## Supplementary information

Supplementary Figure 1

Supplementary Figure 2

Supplementary Figure 3

Supplementary Figure Legends
