# Supplementary figures and images for: Lithium treatment reverses irradiation-induced changes in rodent neural progenitors and rescues cognition
Source: Mol Psychiatry. 2019 Nov 14;26(1):322–40. doi: 10.1038/s41380-019-0584-0 (PMC7815512; doi:10.1038/s41380-019-0584-0)

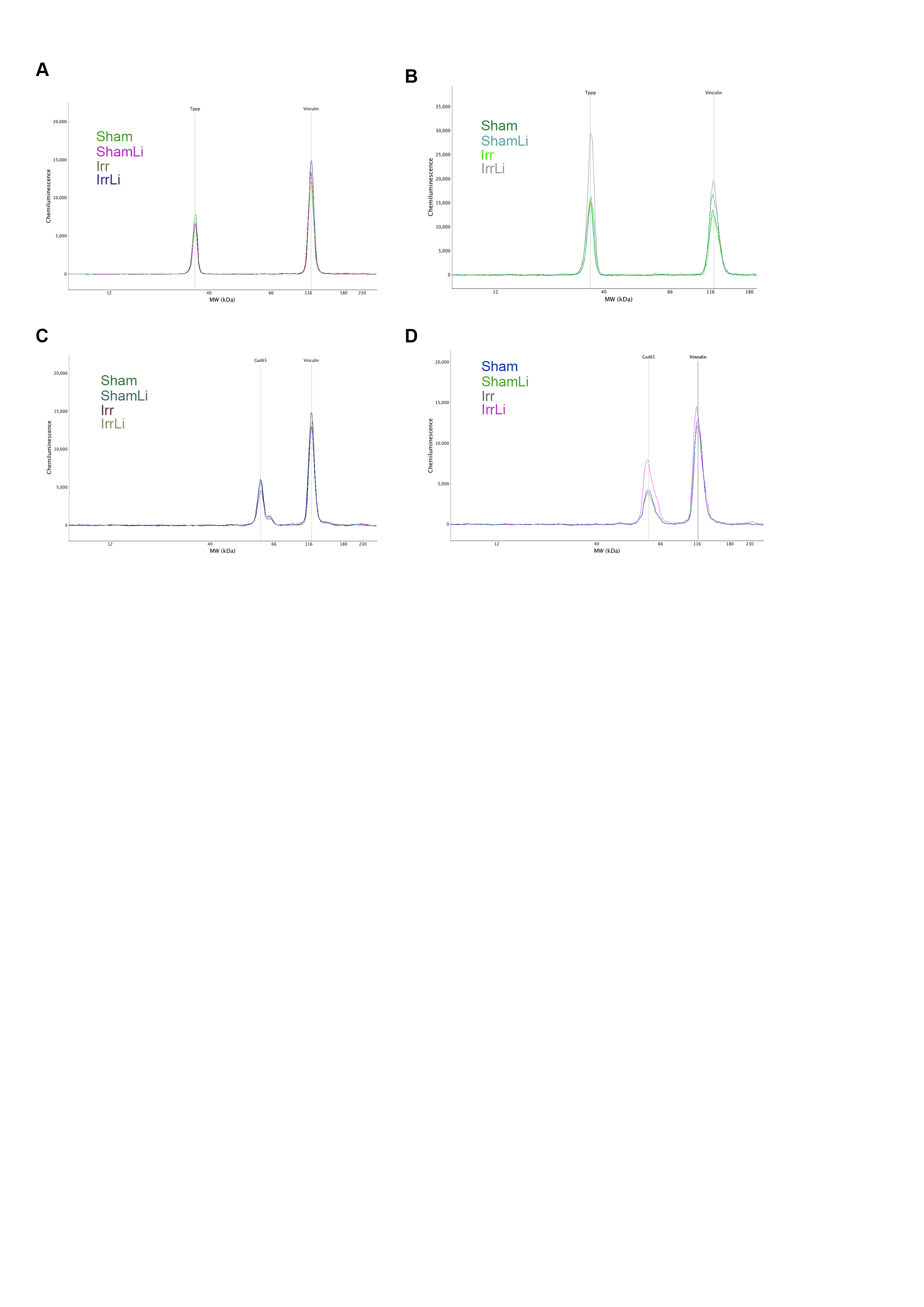

Supplement: Supplementary file 1 — Supplementary Figure 1 [file 41380_2019_584_MOESM1_ESM.tif]

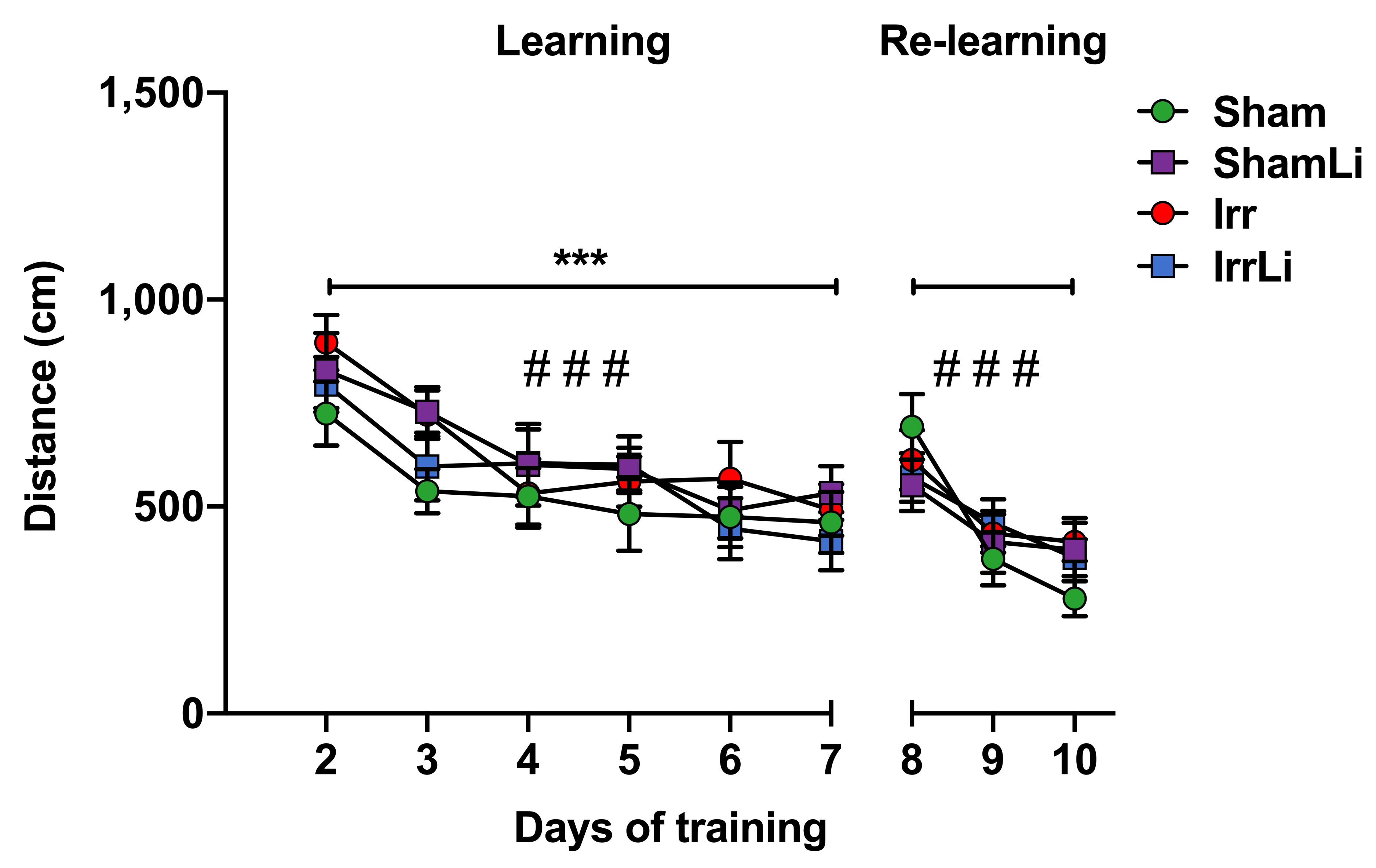

Supplement: Supplementary file 2 — Supplementary Figure 2 [file 41380_2019_584_MOESM2_ESM.tif]

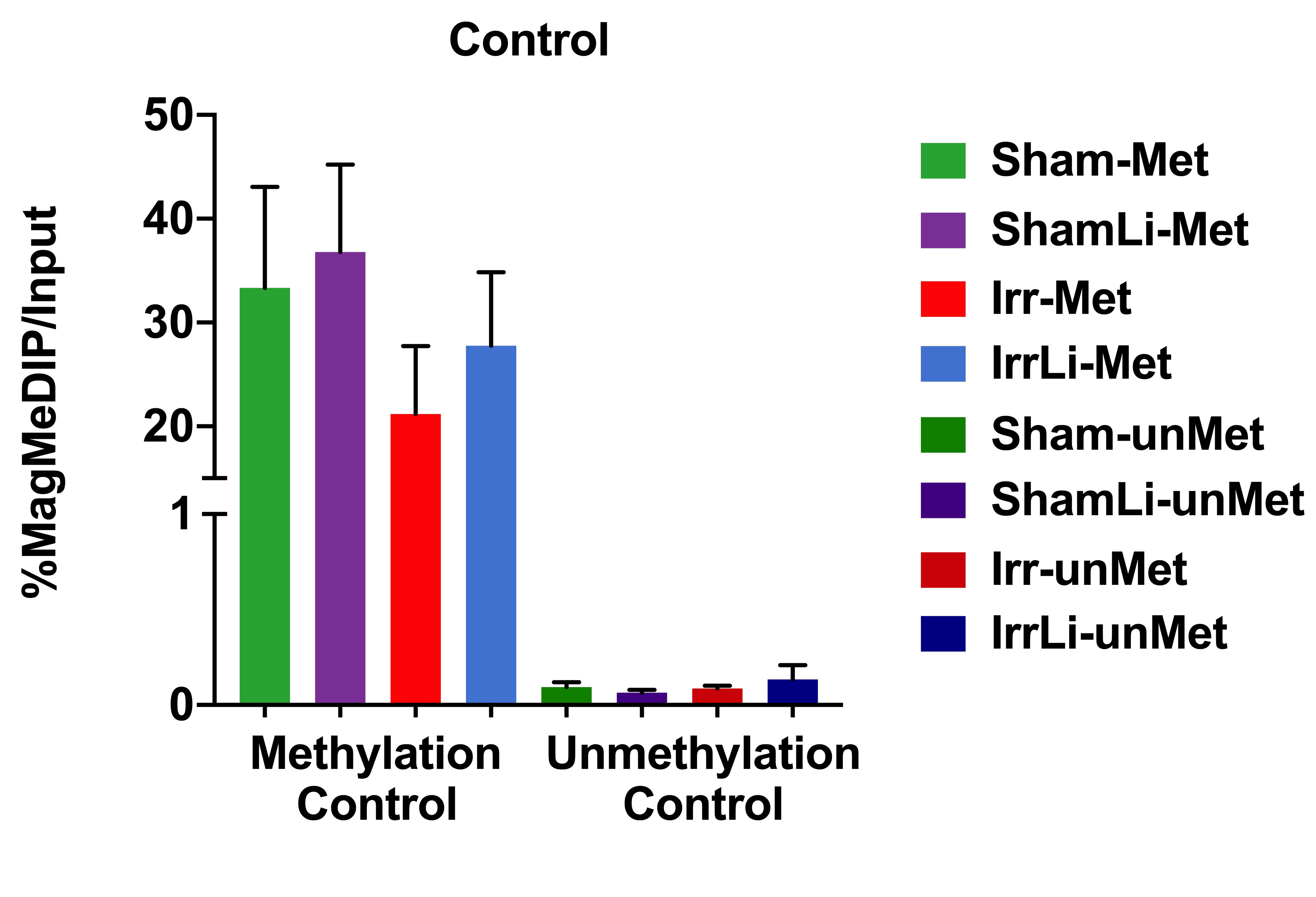

Supplement: Supplementary file 3 — Supplementary Figure 3 [file 41380_2019_584_MOESM3_ESM.tif]
