# Supplementary material for: Lithium treatment reverses irradiation-induced changes in rodent neural progenitors and rescues cognition
Source: Mol Psychiatry. 2019 Nov 14;26(1):322–40. doi: 10.1038/s41380-019-0584-0 (PMC7815512; doi:10.1038/s41380-019-0584-0)
Supplement: Supplementary file 4 — Supplementary Figure Legends [file 41380_2019_584_MOESM4_ESM.docx]

Supplementary Figure 3. DNA immunoprecipitation efficiency. The efficiency of methyl DNA immunoprecipitation of the internal DNA controls of rat NSPCs included in the MeDIP assay (n = 4 - 5). For methylated control (Met), more than 20% of methylation / Input were detected for all groups (Left 4 groups). For unmethylated control (unMet), no methylated signals / Input were obtained for all groups (Right 4 groups).

Supplementary Figure 2. Distance moved during learning and reversal learning in the Morris water maze. All groups successfully learned to find the hidden platform over time in both learning (GEE: W5,270=55.880, ###p<0.001) and reversal learning test (GEE: W2,135 =34.828, ###p<0.001), but the distance moved by the IrrLi and Sham mice was shorter compared to that by Irr and ShamLi mice during the first acquisition period (days 2-7) (GEE group effect: W= 33.213, ***p<0.001; post hoc tests: Sham vs. ShamLi p<0.001, Sham vs.Irr p<0.001, IrrLi vs. ShamLi p=0.003, IrrLi vs. Irr p<0.001). Data are shown as mean+SEM. ###p<0.001.

Supplementary Figure 1. Chemiluminescence signal in the Wes Simple protein quantification (A) Representative chemiluminescence graph showing the peak of both Tppp and Vinculin for each treatment group at PND77. (B) Representative chemiluminescence graph showing the peak of both Tppp and Vinculin for each treatment group at PND91. (C) Representative chemiluminescence graph showing the peak of both GAD65 and Vinculin for each treatment group at PND77. (D) Representative chemiluminescence graph showing the peak of both GAD65 and Vinculin for each treatment group at PND91.
